# Validation of a score tool for measurement of histological severity in juvenile dermatomyositis and association with clinical severity of disease

**DOI:** 10.1136/annrheumdis-2013-203396

**Published:** 2013-09-24

**Authors:** Hemlata Varsani, Susan C Charman, Charles K Li, Suely K N Marie, Anthony A Amato, Brenda Banwell, Kevin E Bove, Andrea M Corse, Alison M Emslie-Smith, Thomas S Jacques, Ingrid E Lundberg, Carlo Minetti, Inger Nennesmo, Elisabeth J Rushing, Adriana M E Sallum, Caroline Sewry, Clarissa A Pilkington, Janice L Holton, Lucy R Wedderburn, the UK Juvenile Dermatomyositis Research Group

**Affiliations:** 1 Rheumatology Unit,UCL Institute of Child Health,London, UK; 2 Department of Health Services Research and Policy, London School of Hygiene and Tropical Medicine, London, UK; 3 Department of Neurology, School of Medicine, University of São Paulo, São Paulo, Brasil; 4 Department of Neurology, Brigham and Women's Hospital and Harvard Medical School, Boston, Massachusetts, USA; 5 Department of Neurology, Children's Hospital of Philadelphia University of Pennsylvania, Philadelphia, Philadelphia, USA; 6 Department of Pathology, Cincinnati Children's Hospital Medical Center, Cincinnati, Ohio, USA; 7 Department of Neurology, Johns Hopkins University School of Medicine, Baltimore, Maryland, USA; 8 Department of Neurology, Mayo Clinic, Rochester, Minnesota, USA; 9 Neural Development Unit, Institute of Child Health UCL, London, UK; 10 Rheumatology Unit, Department of Medicine, Karolinska University Hospital, Karolinska Institute, Stockholm, Sweden; 11 Neuromuscular Disease Unit, Gaslini Institute, Genoa, Italy; 12 Department of Pathology, Karolinska University Hospital, Stockholm, Sweden; 13 Institute for Neuropathology, University of Zurich, Zurich, Switzerland; 14 Pediatric Rheumatology Unit, Department of Pediatrics, School of Medicine, University of São Paulo, São Paulo, Brasil; 15 Dubowitz Neuromuscular Centre, Institute of Child Health UCL, London, UK; 16 Rheumatology Unit, Great Ormond Street Hospital NHS Foundation Trust, London, UK; 17 Department of Molecular Neuroscience, MRC Centre for Neuromuscular Diseases, UCL Institute of Neurology, UCL, London, UK

**Keywords:** Disease Activity, Inflammation, Dermatomyositis

## Abstract

**Objectives:**

To study muscle biopsy tissue from patients with juvenile dermatomyositis (JDM) in order to test the reliability of a score tool designed to quantify the severity of histological abnormalities when applied to biceps humeri in addition to quadriceps femoris. Additionally, to evaluate whether elements of the tool correlate with clinical measures of disease severity.

**Methods:**

55 patients with JDM with muscle biopsy tissue and clinical data available were included. Biopsy samples (33 quadriceps, 22 biceps) were prepared and stained using standardised protocols. A Latin square design was used by the International Juvenile Dermatomyositis Biopsy Consensus Group to score cases using our previously published score tool. Reliability was assessed by intraclass correlation coefficient (ICC) and scorer agreement (α) by assessing variation in scorers’ ratings. Scores from the most reliable tool items correlated with clinical measures of disease activity at the time of biopsy.

**Results:**

Inter- and intraobserver agreement was good or high for many tool items, including overall assessment of severity using a Visual Analogue Scale. The tool functioned equally well on biceps and quadriceps samples. A modified tool using the most reliable score items showed good correlation with measures of disease activity.

**Conclusions:**

The JDM biopsy score tool has high inter- and intraobserver agreement and can be used on both biceps and quadriceps muscle tissue. Importantly, the modified tool correlates well with clinical measures of disease activity. We propose that standardised assessment of muscle biopsy tissue should be considered in diagnostic investigation and clinical trials in JDM.

## Introduction

The idiopathic inflammatory myopathies are rare complex chronic inflammatory disorders affecting muscle, skin and other organs. The most common childhood idiopathic inflammatory myopathy (onset before 16th birthday)—juvenile dermatomyositis (JDM)—has an incidence of 2–3 cases/million/year.^1^ ^2^ The rarity of JDM makes recognition and assessment challenging for clinicians and histopathologists. Until now there has been no standardised histological approach to the assessment of the severity of abnormalities in muscle biopsy specimens from patients with suspected JDM. The JDM Cohort and Biomarker study collects clinical data and samples, including biopsy material, from children with myositis from across the UK and Ireland.^3^

The International Juvenile Dermatomyositis Biopsy Consensus Group has previously designed and tested a scoring tool for assessment of the severity of pathological change in biopsy specimens from patients with suspected or proven JDM.^4^ This tool assesses features agreed to be characteristic of JDM, organised into four domains (inflammatory, vascular, muscle fibre and connective tissue). The tool also includes an overall score of severity, scored by marking a Visual Analogue Score (histopathologists’ VAS) (1.0–10.0 cm). If particular items assessed within a score tool correlate well with clinical features, disease course or response to treatment, the tool would be a valuable addition to the evaluation of this complex disease. A similar approach to quantify features of renal allograft rejection was refined, validated and tested to produce the Banff scoring system.^5^ Retrospective studies of JDM biopsies have suggested that morphological features may correlate with clinical course but standardised scoring systems have not been previously used.^6^ To our knowledge there are no similar standardised tools available for assessment of pathological features in muscle biopsies.

The aims of this study were to reassess the reliability of the JDM score tool in quadriceps, including a comparison with our previous results,^4^ and to assess reliability of the tool when applied to biceps humeri, since this muscle is regularly sampled in some centres. To support the utility of the tool for multicentre studies we wished to determine the intraobserver agreement of the tool elements. Finally, we evaluated whether items of the score tool are associated with clinical measures of disease severity.

## Patients and methods

### Patients and biopsy material

Patients were recruited in the UK (through the UK JDM Cohort and Biomarker study) and Brazil. Both studies had full approval from ethical review boards and were carried out according to the declaration of Helsinki. Criteria for inclusion were that children had definite or probable JDM according to the Bohan and Peter criteria,^7^ and biopsy material was available for research. All children had disease duration of <12 months before biopsy and had their biopsy sample taken before use of steroids or disease-modifying agents such as methotrexate or other immunosuppressive agents. A total of 55 cases were available: 33 from UK, 22 from Brazil (table 1). UK muscle samples were all from the quadriceps femoris (vastus lateralis): 11 of these were reported in a previous study.^4^ In this study those 11 were analysed only for the correlation with clinical data. Brazil tissues were all from biceps humeri. We have shown that biceps and quadriceps have subtle differences in fibre size, fast:slow fibre ratio and capillary:fibre ratio, therefore the muscle source of biopsy tissue is an important consideration on assessment.^9^

**Table 1:** Patient demographics and clinical features at time of biopsy

			Quadriceps (n=33)	Biceps (n=22)	p Value
Patient characteristic/clinical features					
Age at biopsy (years), median (IQR)			6.2 (3.3, 10)	7.5 (6.0, 9.3)	0.37
Age at onset (years), median (IQR)			5.7 (3.3, 9.5)	7.1 (5.7, 9.1)	0.31
Gender, female, number (%)			21 (64)	17 (77)	0.36
Time between symptoms onset to biopsy (months), median (IQR)			3.0 (2.0, 6.5)	2.5 (1.9, 5.4)	0.17
Strength of knee extensors by MMT,* n (%)	score	2	5 (21)	4 (18)	
		3	8 (33)	8 (36)	0.238
		4	7 (29)	10 (46)	
		5	4 (17)	0 (0)	
Strength of elbow flexion by MMT,* n (%)	score	2	2 (9)	4 (18)	
		3	10 (43)	8 (36)	0.273
		4	6 (26)	9 (41)	
		5	5 (22)	1 (5)	
CMAS,† median (IQR)			23.5 (13.3–36.8)	N/A	N/A
PGA,‡ median (IQR)			5.9 (3.5–7.2)	N/A	N/A
Calcinosis, n (%)			0 (0)	6 (27)	0.002
Skin ulceration,** n (%)			4 (13)	3 (14)	0.903
Lung involvement,** n (%)			8 (25)	5 (23)	0.848
Cardiac involvement,** n (%)			0 (0)	2 (9)	0.161
Gastrointestinal involvement,** n (%)			10 (31)	9 (41)	0.465

*MMT, manual muscle testing using the Medical Research Council scale, possible scores from 0 to 5.^8^

**Data available on 32 of the 33 cases.

†CMAS, Childhood Myositis Assessment Score, range 0–53.

‡PGA, physicians global assessment, range 0.0–10.0.

In both cohorts, clinical data at disease onset, serum muscle enzyme levels, erythrocyte sedimentation rate and muscle strength measured by manual muscle testing on the Medical Research Council (MRC) scale 0–5 were recorded.^8^ Complications, including calcinosis, skin ulceration, lung, cardiac and gastrointestinal (GI) involvement, were assessed before biopsy. In the UK cohort, data on the Childhood Myositis Assessment Score (CMAS), an assessment of overall strength and stamina^10^ as well as physicians global assessment (PGA, range 0.0–10.0) were also available.^11^

### Histology and immunohistochemistry

Muscle biopsy sampling, histological staining and immunohistochemistry were carried out as described previously.^4^ Histological staining included haematoxylin and eosin, Gomori's trichrome, ATPase pH 4.6 and pH 9.4, nicotinamide adenine dinucleotide dehydrogenase-tetrazolium reductase and acid phosphatase. For immunohistochemistry, primary antibodies used were: anti-human CD3 (UCHT1), anti-human CD68 (KP1), anti-human major histocompatibility complex (MHC) class I heavy chain (W6/32), anti-human neonatal myosin (WB-MHCn) (all from Novacastra, Newcastle-Upon-Tyne, UK) and anti-human CD31 (JC70A, 1/20) (Dako, Cambridgeshire, UK).

### Scoring exercises

The International Working Group on JDM Biopsy previously proposed a score tool for assessment of JDM biopsy, designed using samples from quadriceps femori.^4^ In this study the same group of experts reconvened to assess inter- and intraobserver reliability of the tool, its reliability when used to score biceps tissue samples and to test correlation between elements of the score tool and clinical features. All validation and reliability data were generated from 44 new cases not used in our previous study. For the main scoring exercise to assess inter-observer reliability, 11 quadriceps samples and 11 biceps samples were selected to include cases in each group demonstrating a range of features and severity (judged by HV and JLH). The quadriceps and biceps samples were allocated by a 11×11 Latin square design for each group, as described previously.^4^

A further 22 additional biopsy samples (11 quadriceps, 11 biceps) were each assessed by five scorers, randomly assigned using a separate partial Latin square design (11×5) for quadriceps and biceps. Scorers did not know to which set of results their scores would contribute. Data from 11 quadriceps cases were available from our previous study.^4^ These data allowed inclusion of all 55 cases in the final analysis for association with clinical features. To assess intraobserver agreement, eight quadriceps cases were scored again by eight scorers in an 8×8 Latin square, 3 days apart from the initial scoring exercise. For each scoring exercise the full panel of stained sections was available as above; scorers were aware of age at time of biopsy and the muscle source of each biopsy.

### Data analysis, statistics and decision on most informative items

Data from the scoring exercises were analysed to provide two summary measures, as used previously.^4^ We used an intraclass correlation coefficient (ICC) as a measure of reliability, and as a measure of scorer agreement we used the ratio of the estimates of the SD attributable to the scorer:the SD attributable to the cases (α).^11^ The ICC and α value for each domain and each item were used to classify the data as good, good* or poor.^4^ ^11^ Items reaching an ICC>0.6 were considered to have high reliability while items with an α score<0.4 had high agreement. Where both reliability and agreement were high (ICC>0.6, α<0.4), the item was classified as good; where either reliability or agreement were high, but not both, performance of the item was classified as good*. Where agreement and reliability were low (ICC<0.6, α>0.4), the item was classified as poor. In the intrarater exercise we calculated proportional agreement (pA; the number of exact agreements of score divided by the number of biopsies (n=8)) achieved by each scorer and for each item of the tool. The median (and range) pA across all scorers for each element of the tool is reported.^12^

To explore associations between clinical measures of disease severity and tool items we used the modal score for each item in the tool, with the exception of domain totals for which we used the median values. Examination of these associations was restricted to tool items which consistently exhibited good* or good rating. Specifically, if they achieved good or good* in our original scoring exercise^4^ and in both the 11×11 scoring exercises conducted for this study, they were considered ‘informative items’ and suitable for further analysis. Comparisons of ordered categorical and binary variables (eg, MRC score, presence/absence of skin ulceration, biopsy score tool items) were compared between biopsy groups using Pearson's χ^2^ test or Fisher's exact test, as appropriate. Age at onset, age at biopsy, time to biopsy, histopathologists’ VAS and modified domain total scores were compared using the Mann–Whitney U test.

The scores for the informative items, modified domain total scores and histopathologists’ VAS were assessed for associations with measures of muscle strength by calculating the Spearman's rank correlation coefficient and conducting a test of independence. Pearson's χ^2^ test or Fisher's exact test were used to assess whether scores for informative items were associated with the presence of periungual erythema, skin ulceration, lung or GI involvement. The Kruskal–Wallis test was used to assess whether the modified domain total scores were associated with the presence of periungual erythema, skin ulceration, lung or GI involvement. This test was also used to assess whether the scores for the six informative items were associated with PGA or CMAS in the UK cohort only. Spearman's rank correlation coefficient was used to assess correlation between modified domain total scores and PGA, modified domain total scores and CMAS, histopathologists’ VAS and PGA and CMAS. All p values reported are unadjusted for multiple testing.

## Results

### Clinical data

Fifty-five patients with JDM (38 female, 17 male) were included in this study. Table 1 shows the patient demographic and clinical data. Patients had a median age at onset of 6.42 years (IQR 4.04–9.13) and median disease duration of 3.0 months (IQR 2–6) at time of biopsy. There were no significant differences in age at biopsy, duration of disease before biopsy or clinical severity between the two groups of patients, with one exception: at the time of biopsy, the Brazil cohort had six (27%) cases with calcinosis, while the UK cohort had none (p=0.002). Proximal muscle strength as measured by manual muscle testing did not differ between the two groups. CMAS and PGA data were available only from the UK cohort.

### Score tool reliability

The score tool and accompanying instructions are shown in online supplementary table S1.^4^ Data on score tool reliability were generated from 22 cases (11 quadriceps, 11 biceps), all new cases compared with our previous study.^4^ Overall scores for inflammatory and muscle fibre domains, as well as several items from each of these domains and severity assessment by histopathologists, reached high reliability for both quadriceps and biceps samples (table 2). These items were also reliable in our previous study.^4^ Intrarater agreement, assessed by pA, was substantial or better (>0.6) in all but one element. The median pA was ≥75% for all the informative items (see online supplementary data, table S2).

**Table 2:** Results of intraclass coefficient (ICC) and measure of agreement (α score) for both sets of 11 biopsies* scored in the 11×11 scoring exercises, with 95% CIs†

Domain	Quadriceps		Biceps	
	ICC	α Score	ICC	α Score
Inflammatory domain	**0.81 (0.61 to 0.93)**	**0.38 (0.19 to 0.74)**	**0.80 (0.62 to 0.93)**	**0.34 (0.16 to 0.68)**
**CD3+ endomysial infiltration**	**0.72 (0.53 to 0.89)**	**0.15 (0.0 to 0.39)**	**0.74 (0.56 to 0.90)**	**0.26 (0.08 to 0.54)**
**CD3+ perimysial infiltration**	**0.61 (0.39 to 0.83)**	**0.31 (0.05 to 0.67)**	**0.75 (0.57 to 0.91)**	**0.20 (0 to 0.44)**
CD3+ perivascular infiltration	0.58 (0.37 to 0.82)	0.41 (0.14 to 0.84)	0.58 (0.37 to 0.82)	0.44 (0.17 to 0.90)
**CD68+ endomysial infiltration**	**0.66 (0.45 to 0.86)**	**0.40 (0.17 to 0.82)**	**0.62 (0.41 to 0.84)**	**0.22 (0.0 to 0.53)**
CD68+ perimysial infiltration	0.48 (0.27 to 0.76)	0.59 (0.24 to 1.2)	**0.79 (0.63 to 0.93)**	**0.18 (0.0 to 0.40)**
CD68+ perivascular infiltration	0.40 (0.20 to 0.70)	0.83 (0.37 to 1.65)	0.33 (0.15 to 0.63)	0.97 (0.43 to 0.93)
Vascular domain	0.48 (0.27 to 0.76)	0.57 (0.23 to 1.2)	0.49 (0.28 to 0.76)	0.54 (0.21 to 1.10)
**Capillary dropout**	0.39 (0.20 to 0.68)	0.73 (0.29 to 1.47)	0.26 (0.11 to 0.55)	0.95 (0.34 to 2.0)
Arterial abnormality	0.40 (0.21 to 0.70)	0.56 (0.18 to 1.18)	0.42 (0.23 to 0.71)	0.52 (0.15 to 1.09)
**Infarction**	**0 (0 to 0.16)**	**0**	**0.42 (0.22 to 0.71)**	**0.34 (0 to 0.80)**
**Muscle fibre domain**	**0.80 (0.63 to 0.93)**	**0.25 (0.10 to 0.52)**	**0.86 (0.71 to 0.95)**	**0.26 (0.12 to 0.52)**
MHC class I overexpression	**0.31 (0.14 to 0.62)**	0.28 (0.0 to 0.82)	0 (0 to 0.14)	0
**Perifascicular atrophy**	**0.63 (0.43 to 0.85)**	**0.28 (0 to 0.61)**	**0.76 (0.58 to 0.910**	**0.29 (0.11 to 0.59)**
**Neonatal myosin**	**0.83 (0.68 to 0.94)**	**0 (0 to 0.20)**	**0.88 (0.76 to 0.96)**	**0.06 (0 to 0.20)**
Fibre atrophy: non-perifascicular	0.28 (0.13 to 0.59)	0.83 (0.29 to 1.72)	0.32 (0.15 to 0.62)	0.76 (0.26 to 1.56)
**Regeneration/degeneration/necrosis: perifascicular**	**0.58 (0.36 to 0.82)**	**0.39 (0.12 to 0.81)**	**0.84 (0.70 to 0.94)**	**0.18 (0.05 to 0.30)**
Regeneration/degeneration/necrosis: non-perifascicular	**0.51 (0.30 to 0.78)**	**0.38 (0.05 to 0.81)**	0.60 (0.38 to 0.83)	0.50 (0.22 to 1.0)
Internal myonuclei	**0.63 (0.42 to 0.85)**	**0.33 (0.09 to 0.69)**	0.32 (0.15 to 0.63)	0.63 (0.16 to 1.34)
Connective tissue domain	0.34 (0.16 to 0.64)	0.74 (0.27 to 1.52)	0.41 (0.21 to 0.70)	0.62 (0.23 to 1.28)
Any endomysial fibrosis	0.35 (0.17 to 0.65)	0.66 (0.22 to 1.38)	0.32 (0.15, 0.63)	0.60 (0.11 to 1.39)
Any perimysial fibrosis	0.19 (0.07 to 0.47)	1.03 (0.26 to 2.17)	0.36 (0.18 to 0.66)	0.70 (0.26 to 1.44)
**Histopathologists’ Visual Analogue Score for severity**	**0.77 (0.60 to 0.91)**	**0.20 (0.03 to 0.44)**	**0.87 (0.74 to 0.96)**	**0.19 (0.08 to 0.40)**

*All 22 cases used to generate these data were new cases, distinct from the previous study.^4^

†ICC>0.6 indicates high reliability, α score <0.4 indicates high agreement.

‡Items shown in bold reached good or good*, as detailed in text.

**Figure 1:**
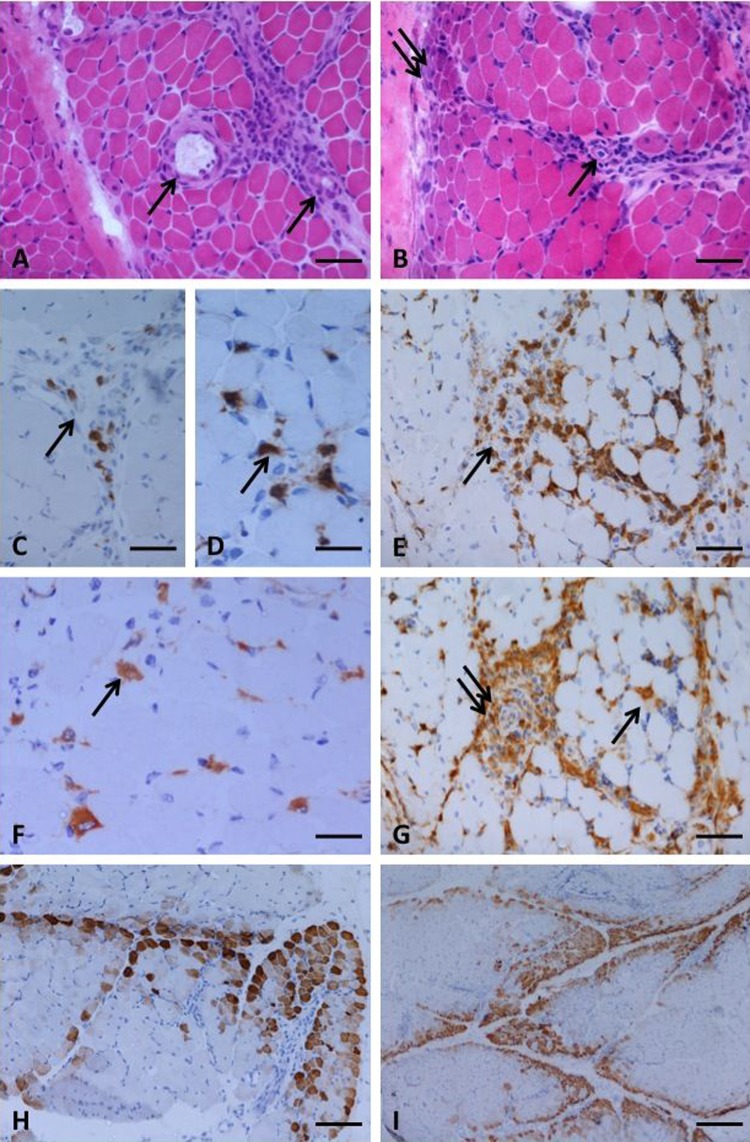
Features of dermatomyositis including the informative score tool items selected for the modified score tool, illustrated in a quadriceps biopsy (A, C, D, F and H) and in a biceps biopsy (B, E, G and I). Perivascular inflammation was seen, often with a perimysial localisation (A and B, arrows indicate vessels). Perifascicular fibre atrophy was a feature of some biopsies, and other fibre abnormalities including basophilia, indicating regeneration, were often more prominent in perifascicular regions (B, double arrow). CD3 immunoreactive T cells were present in the perimysium (C and E, arrows) and also the endomysium (D, arrow). Macrophage infiltrates were identified by CD68 immunohistochemistry in the endomysium (F and G, arrow) and also around vessels (G, double arrow). Neonatal myosin expression could often be seen to have a characteristic perifascicular pattern (H and I). (A and B) haematoxylin and eosin; (C, D and E) CD3 immunohistochemistry; (F and G) CD68 immunohistochemistry; (H and I) neonatal myosin immunohistochemistry. Bars represent: 50 µm in A, B, C, E and G; 25 µm in D and F; 100 µm in H; 260 µm in I.

Items that can be reliably assessed by the same observer on different occasions and different observers will be useful in future studies. Therefore we limited further analysis to informative items—that is, those that were the most reliable, shown in bold in table 2. Two of these, overexpression of MHC protein on muscle fibres and infarction, had an α score of 0 indicating high agreement, but low variability since they were either always abnormal (MHC overexpression) or very rarely seen (infarction). These items were excluded from the modified score tool. Selection of an element for further analysis depended on the performance of that element rather than the importance of the pathological feature for diagnostic purposes. Representative examples of items selected for inclusion in the modified score tool, from both biceps and quadriceps biopsies, are shown in figure 1.

### Association with disease severity measures

We reasoned that a modified score tool containing the most reliable items would be an appropriate instrument to investigate associations with clinical measures of disease. The most reliable items fell into two domains of the score tool: inflammatory and muscle fibre. Using these items, a modified total score range was calculated for each of these domains. Scores for these informative items, modified domain total scores and overall histopathologists’ VAS score data were analysed for all 55 cases (table 3). Comparison of the number of biopsies scoring high or low for each of these items suggested that the biceps samples showed more severe pathology than quadriceps, with differences between scores for the modified muscle fibre domain total, two individual items in the muscle fibre domain, as well as a significantly higher histopathologists’ VAS for severity in biceps compared with quadriceps (table 3).

**Table 3:** Comparison of tool scores for the informative items used in clinical correlation analysis, in quadriceps and biceps biopsies

Domain		Quadriceps (n=33)	Biceps (n=22)	p Value
Inflammatory domain	Tool score	n (%)	n (%)	
CD3+ endomysial infiltration	0	11 (33)	11 (50)	
	1	14 (42)	9 (41)	0.27
	2	8 (24)	2 (9)	
CD3+ perimysial infiltration	0	11 (33)	12 (55)	
	1	12 (36)	7 (32)	0.22
	2	10 (30)	3 (14)	
CD68+ endomysial infiltration	0	2 (6)	3 (14)	
	1	8 (24)	5 (23)	0.63
	2	23 (70)	14 (64)	
Inflammatory domain total (modified), median (IQR)	Possible range 0–6	4 (1.5, 5)	2.5 (1, 4)	0.15
Muscle fibre domain				
Perifascicular atrophy	0	22 (67)	6 (27)	
	1	0 (0)	1 (5)	0.01
	2	11 (33)	15 (68)	
Neonatal myosin	0	9 (27)	8 (36)	
	1	24 (73)	14 (64)	0.48
Regeneration/degeneration/necrosis: perifascicular	0	20 (61)	6 (27)	
	1	2 (6)	1 (5)	0.04
	2	11 (33)	15 (68)	
Muscle fibre domain total (modified),	Possible range 0–5	2 (0, 4)	5 (1, 5)	0.01
Histopathologists' VAS, median (IQR)	Range 0–10	3.3 (1.1, 5.9)	6.1 (2.2, 7.5)	0.023

VAS, Visual Analogue Score.

There was evidence to suggest that measures of weakness were associated with biopsy scores for all of the informative items, the modified total domain scores and the histopathologists’ overall severity score (table 4). Specifically, a higher modified total for both domains was strongly associated with elbow flexor strength score as assessed by the MRC scale (0–5), r=−0.59 p<0.0001: r=−0.60 and p<0.0001 for inflammatory and muscle fibre domains, respectively. Within the muscle fibre domain substantial correlations were seen between the neonatal myosin positivity and both measures of strength (r=−0.57 p<=0.001). The histopathologists’ VAS was also significantly associated with measures of weakness (table 4). No associations were found between the six informative items and periungual erythema, skin ulceration, lung or GI involvement (data not shown).

**Table 4:** Associations between manual muscle testing (MMT) and items of the modified score tool for the combined cohort (London and Brazil)

Domain and item	Knee extensor MMT		Elbow flexion MMT	
	r*	p Value†	r*	p Value†
Inflammatory domain				
CD3+ endomysial infiltration	−0.40	0.006	−0.44	0.003
CD3+ perimysial infiltration	−0.40	0.007	−0.41	0.006
CD68+ endomysial infiltration	−0.53	0.002	−0.62	<0.001
Inflammatory domain total (modified)	−0.56	0.001	−0.59	<0.0001
Muscle fibre domain				
Perifascicular atrophy	−0.30	0.040	−0.40	0.006
Neonatal myosin	−0.57	0.001	−0.57	<0.001
Regeneration/degeneration/necrosis: perifascicular	−0.38	0.009	−0.53	0.002
Muscle fibre domain total (modified)	−0.45	0.002	−0.60	<0.0001
Histopathologists’ Visual Analogue Score for severity	−0.45	0.002	−0.62	<0.0001

*Spearman's rank correlation coefficient.

†For test of independence.

For quadriceps biopsies, where data on PGA and CMAS were also available, PGA was associated with the biopsy score for the informative items in the inflammatory domain (CD3+ endomysial, CD3+ perimysial and CD68+ endomysial) and two items in the muscle fibre domain (neonatal myosin and perifascicular regeneration/degeneration/necrosis). Both modified domain total scores were moderately correlated with PGA, with the inflammatory domain showing a stronger relationship. In all of the above the direction of the association was as expected; a higher biopsy feature score was associated with higher PGA. Both modified total muscle fibre and modified total inflammatory domains were weakly correlated with CMAS. Details of these correlations are shown in online supplementary table S3.

## Discussion

These data provide the first validation of a histological score tool estimating severity in JDM, much needed in this uncommon but potentially devastating autoimmune childhood disease. The tool is designed to measure histological severity using semiquantitative assessment of histological features, rather than to diagnose the condition. This study extends our earlier findings and demonstrates the reliability of the tool, with low inter- and intraobserver variability. Importantly, the most reliable items of the scoring system correlate well with measures of clinical disease activity.

Our study used cases from two different countries, where the muscle used for diagnostic biopsy differs. Although all biopsies were taken early in disease course, calcinosis was more common in cases from Brazil, perhaps reflecting disease severity in that cohort,^13^ and biceps biopsy samples were also scored as more severe in several items (table 3). As biceps samples were not available from UK cases, nor quadriceps tissue from Brazilian cases and no case had samples from both muscles, it was not possible to test how the site of the biopsy affects pathological change. It is also possible that there are other differences between the groups of patients, related not to biopsy site, but to differences in clinical care, ethnicity or environment. Despite these potential confounders we found that the score tool functioned equally well on biceps and quadriceps tissue, and the same score items were the most reliable for both sample sets. By incorporating biceps samples we have generated data suggesting that the score tool can be applied to a muscle other than quadriceps. This provides confidence for inclusion in future studies of centres whose biopsy site is routinely either biceps or quadriceps.

After identifying morphological features that proved reliable between different assessors and different muscles, we showed that these items were moderately or strongly correlated with muscle strength, and with the overall PGA and CMAS, where available. Thus the score tool appears to correlate well with muscle disease activity. A limitation to this analysis is that skin score data were not available on a sufficiently large number of cases to compare biopsy assessment with skin disease activity.

The adoption of agreed protocols for histological assessment of tissue has provided important progress in other diseases, especially in conditions where semiquantitative analysis of specific features has been found to correlate with clinical severity and hence influence management. For example the Banff scoring of renal pathology is widely used to quantify allograft rejection, in trials of anti-rejection drugs and in clinical practice. This system has been refined, altered, validated and tested in several stages.^5^ Similarly, the BrainNet Europe consortium has tested, standardised and validated assessment of features such as α-synuclein immunoreactive structures and amyloid β, in neurodegenerative diseases.^14^ ^15^

In JDM, some evidence suggests that histopathological features indicative of vasculopathy correlate with more aggressive disease,^16^ or that features of vasculopathy and necrosis may predict chronicity.^6^ However, those studies did not include biopsy analysis by a large group of observers and it is therefore difficult to assess how readily they would translate to multiple centres. One difficulty with assessment of rare diseases is ensuring adequate training for pathologists who may encounter only occasional cases. Online image databases might assist with this problem. To circumvent technical barriers and ensure that the tool is robust we have chosen to select features that were most reliable between a group of assessors and used standard, widely available, histopathological stains in preparation of sections.

A limitation of our study is the large number of hypothesis tests conducted to evaluate associations between biopsy features and measures of disease severity. This is likely to result in a high false discovery rate and therefore the reported p values must be interpreted with caution. However, the consistency of the data for associations with muscle strength, warrant consideration and further investigation.^17^ ^18^

Long-term prospective studies are needed to test whether the JDM muscle score tool (and which items of the tool), using tissue obtained at the time of diagnosis, correlates well with disease course, treatment response or disease complications. It will be interesting to test how specific aspects of the tool correlate with more recently reported biomarkers, including the type I interferon gene signature, serum chemokine score or plasmacytoid denritic cells.^19–22^

In conclusion we have shown for the first time that a modified JDM biopsy score tool has high inter- and intraobserver agreement and can be used on both biceps and quadriceps muscle tissue. We suggest that inclusion of this simple, semiquantitative measure into routine diagnostic investigation and clinical trials in children with JDM, should facilitate a more standardised comparison of cases between studies and different centres.

## Supplementary Material

Web supplement 1
