# Supplementary material for: Validation of a score tool for measurement of histological severity in juvenile dermatomyositis and association with clinical severity of disease
Source: Ann Rheum Dis. 2013 Sep 24;74(1):204–10. doi: 10.1136/annrheumdis-2013-203396 (PMC4283618; doi:10.1136/annrheumdis-2013-203396)
Supplement: Web supplement 1 [file annrheumdis-2013-203396-s1.pdf]

Varsani et al, 2013: SUPPLEMENTARY DATA

Supplementary Table S1A: The JDM Biopsy Score tool as used

JDM BIOPSY SCORE TOOL

Refer to detailed instructions below for definitions

Case identifier		Date scored	
Date of Biopsy		Gender (M/F)	
Research code		Age at biopsy	

DOMAIN	Score options	SCORE HERE	NOTES
**INFLAMMATORY DOMAIN**			
CD3+ endomysial infiltration	0, 1, 2		
CD3+ perimysial infiltration	0, 1, 2		
CD3+ perivascular infiltration	0, 1, 2		
CD68+ endomysial infiltration	0, 1, 2		
CD68+ perimysial infiltration	0, 1, 2		
CD68+ perivascular infiltration	0, 1, 2		
**VASCULAR DOMAIN**			
Capillary dropout	0 (N) or 1 (Y)		
Arterial abnormality	0 (N) or 1 (Y)		
Infarction	0 (N) or 1 (Y)		
**MUSCLE FIBRE DOMAIN**			
MHC Class I over- expression	0 (N) or 1 (Y)		
Perifascicular atrophy	0, 1, 2		
Neonatal myosin	0 or 1		
Fibre atrophy: non perifascicular	0 (N) or 1 (Y)		
Regeneration/ Degeneration /Necrosis: peri-fascicular	0, 1, 2		
Regeneration/ Degeneration /Necrosis: non-peri-fascicular	0, 1, 2		
Internal myonuclei in non-basophilic otherwise normal fibres	0 or 1		
**CONNECTIVE TISSUE DOMAIN**			
Any endomysial fibrosis	0 (N) or 1 (Y)		
Any perimysial fibrosis	0 (N) or 1 (Y)		

VISUAL ANALOGUE SCALE - OVERALL IMPRESSION OF ABNORMALITY

0 ____ **10**

no abnormality **much abnormality**

Please make a mark on this 10cm line to indicate overall severity from no abnormality to much abnormality; do not write a number.

Supplementary Table S1B Score tool definitions and Instructions

JDM SEVERITY SCORE TOOL: INSTRUCTIONS for USE

DOMAIN	Score options	Definitions and Instructions
CD3+ endomysial infiltration	0, 1, 2	For each of endomysial, perimysial, perivascular distributions, score for CD3+ infiltrating cells as follows: if none, or less than 4 cells in x20 field - score 0; if ≥4 cells in a x20 field and/or 1 cluster (where a cluster is approx 10 cells or more) - score 1; if ≥2 clusters in whole biopsy, and/or diffusely infiltrating cells (ie ≥20 cells in a 20x field) - score 2
CD3+ perimysial infiltration	0, 1, 2	
CD3+ perivascular infiltration	0, 1, 2	
CD68+ endomysial infiltration	0, 1, 2	For each of endomysial, perimysial, perivascular distributions, score for CD68+ infiltrating cells as follows: if none, or less than 4 cells in x20 field - score 0; if ≥4 cells in a x20 field and/or 1 cluster (where a cluster is approx 10 cells or more) - score 1; if ≥2 clusters in whole biopsy, and/or diffusely infiltrating cells (ie ≥20 cells in a 20x field) - score 2
CD68+ perimysial infiltration	0, 1, 2	
CD68+ perivascular infiltration	0, 1, 2	
**VASCULAR DOMAIN**		
Capillary dropout (Using immunohistochemistry for CD31)	0 (N) or 1 (Y)	Obvious and marked decrease in the density of capillary network, not restricted to areas of perifascicular atrophy. Absence – N; Presence – Y
Arterial abnormality	0 (N) or 1 (Y)	Mural thickening and/or endothelial swelling and/or transmural inflammation in arteries/arterioles. Absence – N; Presence – Y
Infarction	0 (N) or 1 (Y)	Well demarcated regional loss of muscle fibre nuclei and loss of normal cytoarchitecture. Absence – N; Presence – Y
**MUSCLE FIBRE DOMAIN**		
MHC Class I over- expression	0 (N) or 1 (Y)	Presence of MHC class I staining on or in muscle fibres. Absence – N; Presence – Y
Perifascicular atrophy	0, 1, 2	Affecting ≥ 6 fibres out of 10 along one edge of a fasciculus, not exclusive to type IIb fibres**.** Absent - score 0. Present in one or 2 fascicles - score 1. Present in 3 or more fascicles - score 2
Neonatal myosin (using immunohistochemistry for neonatal myosin)	0 or 1	Less than 6 positive fibres in a x20 field - score 0; ≥ 6 positive fibres in a x20 field - score 1
Fibre atrophy: non-perifascicular	0 (N) or 1 (Y)	Fibre atrophy: non-perifascicular (outside normal variation for age). Absence – N; Presence – Y
Regeneration/ Degeneration /Necrosis: peri-fascicular	0, 1, 2	Includes: focal basophilia within a fibre, vacuolation, myofibrillar rarefaction and/or pallor, myophagocytosis, acid phosphatase positive fibres. For each of perifasciular and non-perifascicular, score as follows. None – score 0. If any of the features in 1 or 2 fasciculi score 1. If any of the features in 3 or more fasciculi score 2
Regeneration/ Degeneration /Necrosis: non-peri-fascicular	0, 1, 2	
Internal myonuclei in non-basophilic otherwise normal fibres	0 or 1	Internal myonuclei in non-basophilic cells (in otherwise normal fibres) in 1 or more fasciculi excluding myotendinous junctions. If < 3% fibres - score 0. If ≥ 3% fibres - score 1
**CONNECTIVE TISSUE DOMAIN**		
Any endomysial fibrosis	0 (N) or 1 (Y)	For fibrosis in each of endomysial and perimysial distributions, score as follows: Absence: score N**.** Presence of any: score Y
Any perimysial fibrosis	0 (N) or 1 (Y)	

Supplementary Table S2: Intra-rater agreement for items of the score tool assessed by proportion of agreement (pA).

	Proportion of Agreement Median* (range)
**Item name**	
**CD3+ endomysial infiltration**	**0.8 (0.5 – 1.0)**
**CD3+ perimysial infiltration**	**0.7 (0.6 – 0.9)**
CD3+ perivascular infiltration	0.8 (0.6 – 1.0)
**CD68+ endomysial infiltration**	**0.8 (0.6 – 1.0)**
CD68+ perimysial infiltration	0.8 (0.6 – 0.9)
CD68+ perivascular infiltration	0.6 (0.5 – 0.8)
Capillary dropout	0.8 (0.5 – 1.0)
Arterial abnormality	0.9 (0.5 – 0.9)
**Perifascicular atrophy**	**0.8 (0.6 - 1.0)**
**Neonatal myosin**	**1.0 (0.8 – 1.0)**
Fibre atropy – no-perifascicular	0.8 (0.5 – 1.0)
**Regeneration/degeneration/necrosis: peri-fascicular**	**0.8 (0.5 – 1.0)**
Regeneration/degeneration/necrosis: non peri-fascicular	0.8 (0.4 – 0.9)
Internal myonuclei	0.9 (0.9 – 1.0)
Endomysial fibrosis	0.9 (0.6 – 1.0)
Perimysial fibrosis	0.8 (0.6 – 1.0)

median evaluated over 8 scorers. Bold indicates informative items, defined as those which achieved good or good in original scoring exercise [4] and in both of the 11x11 scoring exercises conducted for this study

Supplementary Table S3 Correlations between tool items and measures of disease activity Physicians global assessment and childhood myositis assessment score

	PGA		CMAS	
**Domain & Item**	**r**	**p-value**	**r**	**p-value**
**INFLAMMATORY DOMAIN**				
CD3+ endomysial infiltration*	n/a	0.02	n/a	0.16
CD3+ perimysial infiltration*	n/a	0.02	n/a	0.13
CD68+ endomysial infiltration*	n/a	0.01	n/a	0.05
**Inflammatory Domain total**(modified)**	0.61	0.001	-0.43	0.03
**MUSCLE FIBRE DOMAIN**				
Perifascicular atrophy*	n/a	0.23	n/a	0.15
Neonatal myosin*	n/a	0.01	n/a	0.02
Regeneration/ Degeneration /Necrosis: peri-fascicular*	n/a	0.03	n/a	0.18
**Muscle Fibre Domain total**(modified)**	0.40	0.01	-0.41	0.04

* p-value - Kruskall Wallis test, ** p-value - test of independence: r – Spearman's rank correlation coefficient.
n/a – r value not available, since the Kruskall Wallis test does not generate a r value
